# Ultrasensitive Detection of Malachite Green Isothiocyanate Using Nanoporous Gold as SERS Substrate

**DOI:** 10.3390/ma16134620

**Published:** 2023-06-27

**Authors:** Deepti Raj, Noor Tayyaba, Ginevra De Vita, Federico Scaglione, Paola Rizzi

**Affiliations:** Dipartimento di Chimica e Centro Interdipartimentale NIS (Nanostructured Surfaces and Interfaces), Università di Torino, Via Pietro Giuria 7, 10125 Torino, Italy

**Keywords:** chemical dealloying, amorphous alloys, nanoporous gold, SERS, environmental monitoring

## Abstract

In this article, a high-performance nanostructured substrate has been fabricated for the ultrasensitive detection of the organic pollutant, Malachite green isothiocyanate (MGITC), in aquatic systems via the Surface Enhanced Raman Spectroscopy (SERS) technique. The chemical dealloying approach has been used to synthesize a three-dimensional nanoporous gold substrate (NPG) consisting of pores and multigrained ligament structures along thickness. The formation of the framework in NPG-5h has been confirmed by SEM with an average ligament size of 65 nm at the narrower neck. Remarkable SERS performance has been achieved by utilizing the NPG-5h substrate for the detection of MGITC, showing a signal enhancement of 7.9 × 10^9^. The SERS substrate also demonstrated an impressively low-detection limit of 10^−16^ M. The presence of numerous active sites, as well as plasmonic hotspots on the nanoporous surface, can be accredited to the signal amplification via the Localized Surface Plasmon Resonance (LSPR) phenomenon. As a result, SERS detection technology with the fabricated-NPG substrate not only proves to be a simple and effective approach for detecting malachite green but also provides a basis for in situ detection approach of toxic chemicals in aquatic ecosystems.

## 1. Introduction

The rapid development of the chemical sector has been progressively contributing to the production of toxic effluents. An increase in the number, concentrations, and diversity of these harmful organic pollutants in wastewater is leading to serious environmental issues [1]. Once released into the environment, the risks of pathogenesis and carcinogenesis towards human health cannot be ignored. As a result, organic pollutant detection has received increased attention in the last few years and has always been considered as a key point in environmental protection. Organic pollutants are typically produced in manufacturing industries such as textiles, food, pharmaceuticals, pesticides, synthetic dyes, and explosives [2,3,4,5]. A synthetic dye that is frequently used in the textile industry as a coloring agent is malachite green (MG), which is classified as a common organic pollutant [1]. MG and its isothiocyanate derivatives are also used as fungicides and are widely used to prevent and treat a variety of fish infections [2,3]. The release of these highly colored effluents, with their complex and prolonged degradable time and genotoxic properties, poses a severe threat to human health and the ecological environment. Due to this, many European countries, as well as Japan, the United States and China, have restricted the use of MG or representative triphenylmethane dyes [4,5]. From 2003 to the present, hundreds of cases of illegal use of MG and its derivatives have been reported by European Union’s Rapid Alert System for Food and Feed (RASFF). Due to this, the EU Commission set a threshold value for this molecule and its derivate of 2 μg/kg (~5.48 nM/L) in food of animal origin [6].

The urgent need to identify organic pollutants prompted the development of innovative analytical procedures to detect organic compounds from water sources. Many different strategies were proposed, including spectroscopic approaches, such as infrared spectroscopy, UV-vis spectroscopy, Raman spectroscopy, mass spectrometric methods, and electroanalytical methods [7,8,9,10]. The above-mentioned techniques have numerous advantages. But still, they require a complex separation process, sophisticated equipment, and a prolonged detection time [11]. Compared to these techniques, Surface-enhanced Raman spectroscopy (SERS) is simple, quick, sensitive, and non-destructive. SERS has been widely used as an effective detection technique in biomedical research, material science, chemistry, biology, and ultra-sensitive chemical and biological analysis [12]. Basically, SERS uses a nanostructured plasmonic metal substrate that amplifies the Raman scattering signal of the analyte molecule by factors as large as 10^3^–10^9^ [13,14,15,16]. The enhanced Raman scattering is primarily due to local field enhancement near nanostructures of noble metals such as silver and gold via LSPR, and is highly dependent on the size, shape, composition, and arrangement of these plasmonic nanostructures. Additionally, there is a ubiquitous presence of “hotspots” on the nanostructured substrate where surface plasmons are intensely focused on a small volume of a few cubic nanometers at the interface [17].

For improving the SERS technique, an efficient and suitable substrate with a high-enhancement factor (EF) and homogeneous large surface area is required. The effectiveness of the SERS substrate is primarily determined by its sensitivity and selectivity [18]. Therefore, it is essential for the SERS substrate to possess a low-detection limit and a large linear-detection range [19]. Recent efforts to enhance the SERS effect have primarily focused on the creation of active substrates in the form of three-dimensional (3D) nano architectures with metal nanoparticles (NPs) rich in nanogaps, which may generate more suitable and effective hotspots for the creation of strong and tunable EF within the detection volume [20]. Among these metal nanoarchitecture, nanoporous gold (NPG) is a suitable substrate because of its exceptional stability, biocompatibility, and repeatability as well as its incredibly strong and controlled surface plasmon resonance from visible to ultraviolet wavelengths [21]. Dealloying is a popular method to fabricate NPG, which involves the selective dissolution of the less noble elements from a solid solution or an amorphous alloy. During this process, the remnant, more noble metal atoms, simultaneously reorganize in interconnected ligaments by surface diffusion [22,23]. It has also been reported that few of the less noble atoms are incorporated in the newly formed ligaments as impurities, due to the surface diffusion-driven mechanism during dealloying [24]. Their presence within the fine nanoporous morphology has been shown to increase the NPG sample’s overall plasmonic effect and catalytic properties [25,26,27,28]. Overall, it is an extremely facile and effective method to produce high-quality NPG for an array of sensing and catalytic applications.

Scaglione et al. [29] synthesized NPG by chemical dealloying and further anodization in oxalic acid to achieve a bimodal morphology. The NPG and 3 min anodized NPG showed SERS EF of 2 × 10^13^ and 1 × 10^17^, respectively, by achieving 10^−16^ M LOD for 4’4-bipyridine. C. Awada et al. [30] investigated the Ag-Au system and porous gold nanostructure revealing that SERS activity led to selective photon adsorption achieving the EF of 10^5^. Hu et al. [31] studied SERS stability and EF by combining dealloying and magnetron sputtering to produce NPG. The EF was reported to be 10^7^. Hence, the SERS performance can be feasibly enhanced by surface modulation and employing NPG as a SERS active substrate. The NPG surface provides a variety of active sites that intensify the excitation of localized surface plasmons, which has been shown to be a key factor in boosting the SERS performance [32]. Additionally, a recyclable nanostructured Au-based SERS active substrate will increase the output, and reduce costs and environmental issues [33].

To achieve these advantages, many SERS substrates based on nanostructured Au have been designed for the detection of organic pollutants in the environment, such as malachite green and its derivatives, dyes, pesticide residues, bisphenol A, and persistent organic pollutants [34,35,36]. For example, Fu et al. [37] designed a SERS substrate using graphene oxide-gold nanoparticle (GO-AuNPs) hybrids, reaching for a low-detection concentration of 2.5 μM in MG. Tan et al. [38] fabricated a SERS substrate with a detection limit of 1.0 pM, using a 3D TiO_2_ nanorod scaffold coated with silver nanoparticles. However, more research is needed in this field to simplify the fabrication process of the substrate, making it cost-effective while improving the overall SERS performance. Therefore, in the present paper, an advanced SERS sensor is developed by dealloying an amorphous melt-spun ribbon precursor, Au_20_Cu_48_Pd_5_Ag_7_Si_20_. The disruption of the amorphous matrix by dealloying and the reorganization of noble atoms in ligaments by surface diffusion, allows to obtain, from a non-SERS active and flat metallic material, much like the amorphous precursor, a nanostructured NPG substrate that shows increased SERS properties [25,26,27,28].

The synthesis of the amorphous precursor and the dealloying procedure will be described first; the methodology undertaken to conduct SERS studies and data analysis will follow. The produced sensor displays an impressively low limit of detection (LOD) and high EF, behaving as a highly active, sensitive, and efficient substrate capable of detecting ultra-low concentrations of MGITC.

## 2. Materials and Methods

### 2.1. Synthesis of the Nanoporous Gold

A master alloy ingot of composition Au_20_Cu_48_Ag_7_Pd_5_Si_20_ was synthesized by arc-melting pure elements (99.95% to 99.99%) in a Ti-gettered Ar atmosphere by using a Buehler electric arc furnace (Edmund Bühler GmbH, Bodelshausen, Germany). This composition results in a suitable amorphous precursor with good glass-forming ability. The ingot was rapidly solidified by the melt-spinning technique (Edmund Bühler GmbH, Bodelshausen, Germany). The molten alloy, kept in a quartz crucible, was ejected from a 2 mm nozzle onto a copper wheel rotating at 25 m/s in a protective Ar atmosphere, producing 25 µm thick and 2 mm wide amorphous ribbons. The as-quenched Au-based amorphous ribbon was chemically dealloyed using a water bath in 10 M HNO_3_ and 0.5 M HF at 70 °C for 5 h. Afterward, the as-dealloyed sample was rinsed with double distilled water several times, then dried in air and stored in a clean vial till characterization. The sample will be addressed as NPG-5h henceforth. The structure of both the as quenched ribbon and the dealloyed sample was determined using a Panalytical X-pert X-ray Diffractometer in Bragg–Brentano geometry (Panalytical, Almelo, The Netherlands) with a Cu Kα radiation. The indexing of diffraction peaks was performed with the use of X’Pert Highscore software (version 2.2c (2.2.3.)). The surface morphology and cross-section of NPG-5h sample were evaluated by Scanning Electron Microscopy (SEM) (TESCAN, Brno, Czech Republic) coupled with Energy Dispersive X-ray Spectroscopy (EDS) (Oxford Ultim-Max 100, Oxford Instruments, Abingdon, UK) to determine its composition. The average ligament size of the NPG-5h sample was evaluated by measuring 160 ligaments at their narrower neck by using the open-source ImageJ software [25].

### 2.2. Preparation of Probe Molecule Solutions

MGITC was used as the probe molecule to evaluate the SERS activity of the NPG-5h sample. All solutions were prepared with chemical-grade reagents, purchased from Sigma Aldrich, and de-ionized water. To prepare the solutions, the powder of malachite green isothiocyanate (MGITC) was accurately weighed and dissolved in 10 mL of de-ionized water to prepare a concentrated mother solution. Further dilutions were carried out from 10^−10^ to 10^−17^ M. A few centimeters long piece of the NPG-5h sample was cut and immersed in the MGITC solution in an Eppendorf tube for 30 min, starting with the least concentrated solution. After 30 min of immersion, the sample was carefully taken out from the solution and allowed to air dry for further 30 min. This approach allows for the maximum absorption of probe molecules on the surface of the sample. After drying, the sample was placed in the sample holder of the SERS instrument to perform the measurements. The same protocol was followed for each MGITC concentration 10^−16^ M, 10^−14^ M, 10^−12^ M and 10^−10^ M. To avoid contamination, new and separate tips, and Eppendorf tubes were used for each concentration. Renishaw in Via Raman Microscope (Renishaw, Wotton-under-Edge, England) with a 785 nm laser line was used for the SERS investigation of carcinogenic MGITC. The detection of MGITC was set up with a 20 × ULWD objective, 0.5% power, and 3 acquisitions with an acquisition time of 20 s. Experiments have been conducted in triplicates to ensure reproducibility.

To study the SERS signal distribution, a SERS intensity mapping image was also acquired on NPG-5h for all concentrations. Maps were collected on a surface area of 100 × 100 µm^2^ with a step size of 25 µm in both directions, monitoring the characteristic peak of the MGITC at 827 cm^−1^.

### 2.3. Data Analysis

The enhancement factor (EF) of NPG-5h was calculated to estimate its SERS capability. Based on SERS spectra, the peak with the highest intensity at 827 cm^−1^ attributed to C-H skeleton bending was chosen as the representative characteristic peak [39]. To calculate the average EF, we used the peak intensity of 10^−16^ M MGITC molecules on an NPG-5h substrate and of 10^−8^ M MGITC molecules on a flat gold substrate. The SERS EF was calculated as follows:

$$E.F=\frac{I_{SERS}}{C_{SERS}}\times \frac{C_{blank}}{I_{blank}}$$

where *I_SERS_* and *I_blank_* are the intensities of the selected scattering band in the SERS spectrum using NPG-5h as substrate and in the Raman spectrum using a flat gold substrate, respectively; *C_SERS_* (10^−16^ M) and *C_blank_* (10^−8^ M) imply the analyte concentrations used for the SERS substrate and flat gold substrates, respectively.

## 3. Results and Discussion

**Figure 1:**
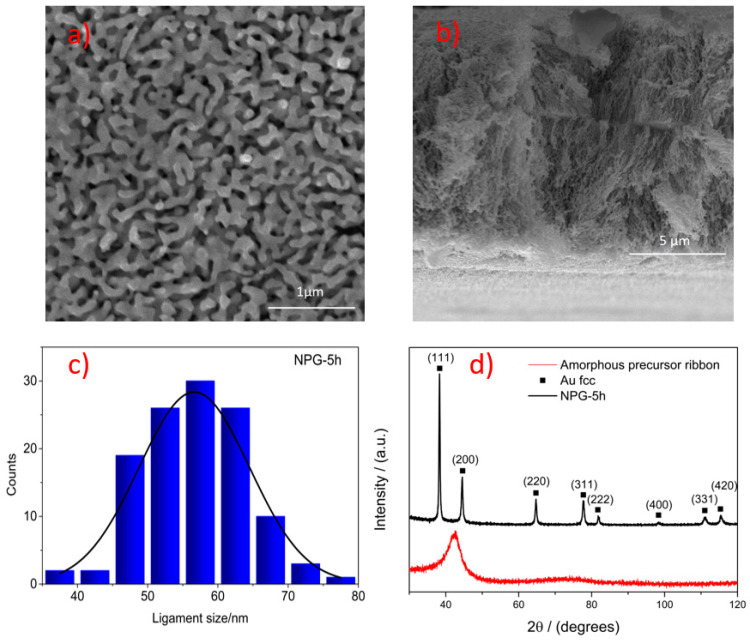
SEM images of the NPG-5h sample: (**a**) top surface view and (**b**) cross-sectional view (**c**) ligament size distribution of NPG-5h (**d**) XRD spectra of NPG-5h (black curve) and amorphous precursor ribbon (red curve).

An analysis of the surface morphology of the dealloyed NPG-5h SERS substrate was conducted using scanning electron microscopy (SEM). The SEM images of the NPG-5h sample surface, as well as the cross-section, are shown in Figure 1a,b. The surface morphology in Figure 1a is dictated by the presence of pores and ligaments. The cross-section in Figure 1b reveals 3D interconnected channels that penetrate the entire ribbon thickness, and after dealloying, it is shrinked from 25 to 12 µm. Figure 1c details the ligament size distribution within the sample surface. The multigrain ligament size, measured at the narrower neck, ranges from 35 to 80 nm and the average ligament size of NPG-5h is estimated to be 65.0 ± 7.5 nm.

Furthermore, EDS analysis (Table 1) reveals Au as the main constituent with minimal residual amounts of other elements, i.e., Si 1.0 at. %, Cu 5.3 at. %, Pd 0.3 at. % and Ag. 0.1 at. %. These residual elements are expected to be trapped in a solid solution inside the Au ligaments, based on the dealloying mechanism, and their distribution can be considered uniform.

The formation of an amorphous alloy, after the melt-spinning synthesis and the formation of the structure after the dealloying process, were effectively validated using XRD analysis. Figure 1d depicts the presence of a single enlarged halo in the diffractogram (in red) related to an amorphous precursor. On the other hand, the diffractogram (in black) for NPG-5h clearly shows the transition of the amorphous phase of the precursor to crystalline with the appearance of typical face-center cubic (fcc) peaks attributed to the Au-rich solid solution. Unlike the catalytic properties that are usually boosted when the catalyst exposes specific crystallographic planes, SERS activity is not affected by planes orientation but the enhanced plasmonic effect is accounted to the morphology of ligaments constituted by a multi-grained crystalline structure, and developed via a different dealloying mechanism of the amorphous precursor. Furthermore, NPG synthesized from amorphous precursors has shown higher SERS activity compared with NPG from crystalline counterparts.

### SERS Analysis

**Figure 2:**
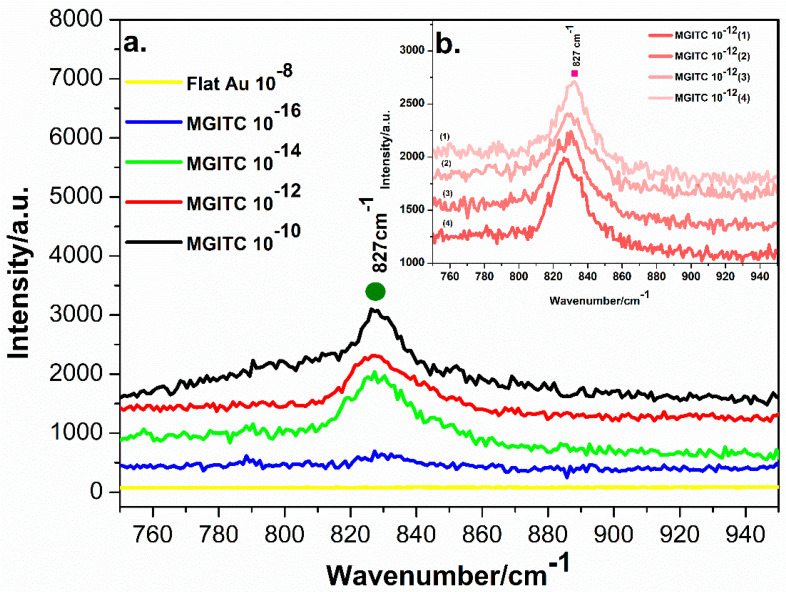
(**a**) SERS spectra for MGITC on NPG-5h obtained in different concentrations, namely 10^−10^ M, 10^−12^ M, 10^−14^ M, 10^−16^ M, and on flat Au in 10^−8^ M. (**b**) Inset of four spectra collected on NPG-5h for MGITC 10^−12^ M.

A SERS analysis was performed to test the sensitivity of NPG-5h for the detection of MGITC. The SERS activity of NPG-5h substrate was measured by using different concentrations of 10^−16^ M, 10^−14^ M, 10^−12^ M and 10^−10^ M MGITC. The spectra obtained for all concentrations, ranging from 750 cm^−1^ to 950 cm^−1^, are shown in Figure 2a. The MGITC spectra shown in Figure 2a were compared with standard Raman spectra for MGITC. The strongest SERS peak was observed at 827 cm^−1^, in agreement with [39] the presence of a high-intensity peak at 806 cm^−1^ and corresponding to the out-of-plane motion of the aromatic hydrogen. It should be also noted that the whole MGITC spectrum slightly differ from the literature [40,41] This variation can be explained by the fact that different substrates inherently have different chemical and morphological characteristics, which can affect the SERS signals. Furthermore, the sample preparation and the measurement conditions, such as laser power, laser spot size, and detector parameters, can also have an influence on the SERS signals [42]. The SERS intensity of MGITC using the NPG-5h was observed to increase, increasing the concentration from 10^−14^ to 10^−10^, as shown in Figure 2a. Moreover, the signal, clearly detected at 10^−14^ M, shows results that are still visible but strongly flattened at 10^−16^ M. Therefore, the limit of detection (LOD) for MGITC in the NPG-5h SERS substrate can be determined as 10^−16^ M, as illustrated in Figure 2a. In Figure 2b (inset of Figure 2a), the SERS signals generated from four random spots collected on the surface after incubation in 10^−12^ M MGITC are reported; the intensity of the peaks, quite similar in each measurement, is proof of the reproducibility of the signal and of the homogeneity of the response of the SERS substrate. The EF was calculated to be 7.9 × 10^9^.

The results of this experiment demonstrate that the SERS detection of MGITC by using the NPG-5h substrate is highly sensitive and effective. This could provide an effective solution to the problem of detection of MGITC in wastewater, thus helping to protect the environment.

**Figure 3:**
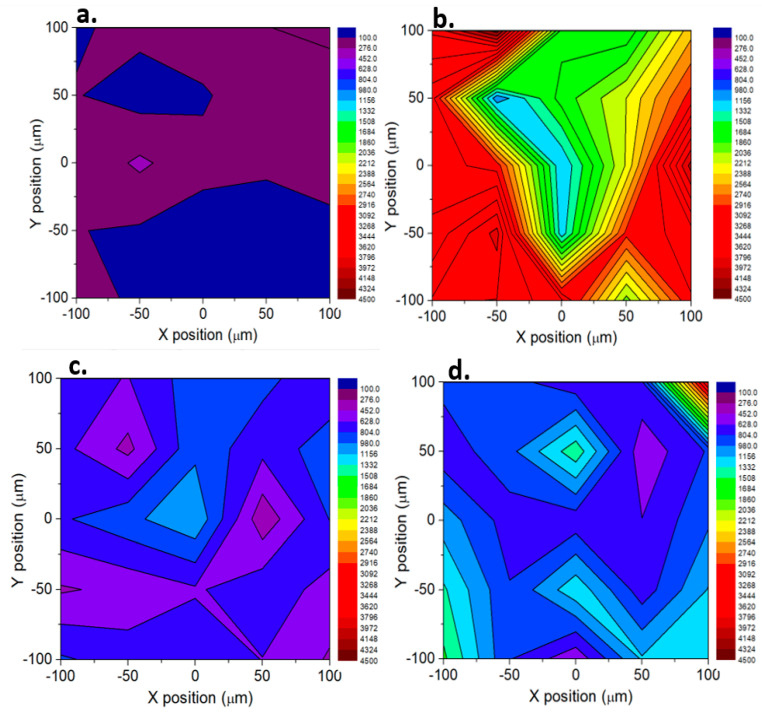
SERS map of MGITC on 100 ×100 µm^2^ area using the NPG-5h as SERS substrate. (**a**) 10^−16^ M concentration of MGITC, (**b**) 10^−14^ M concentration of MGITC, (**c**) 10^−12^ M concentration of MGITC, (**d**) 10^−10^ M concentration of MGITC.

To investigate the influence of the concentration of the MGITC molecules on the SERS signal and the homogeneity of the NPG-5h substrate, a map was acquired per each concentration collecting the SERS signals of 25 sites in a grid of 100 µm × 100 µm; the intensity of the representative signal at 827 cm^−1^ was reported in a colored scale in the maps shown in Figure 3. The observed changes in relative intensity at different MGITC concentrations indicate that the molecules always interact with the NPG-5h surface as represented in Figure 3. At 10^−16^ M (Figure 3a), the dark blue-violet color of the map shows a quite homogeneous response of the substrate when the analyte concentration is extremely diluted and only a few molecules are under the laser spot area. Literature reports that, as the concentration increases, MGITC molecules will occupy all or most of the SERS hot spots on NPG ligaments. Once all sites have been occupied, the molecules that did not directly bond to the surface or in its proximity do not contribute to the SERS signal, therefore the SERS signal intensity is saturated [43]. That situation might be the case for the map of Figure 3b, where we observed remarkable SERS signals for concentrations of 10^−14^ M MGITC; it could be inferred that, at this molecule’s concentration, the substrate is in the conditions of having the surface almost completely occupied with MGITC and, therefore, strong SERS signals are observed. When the concentration is further increased, i.e., 10^−12^ M and 10^−10^ M (Figure 3c,d), the active sites are already saturated, and the intensity is depressed with respect to the previous concentration but higher than the lower one. Nevertheless, random hot spots might contribute to a higher signal, as observed in the upper right corner of Figure 3d. The intensity values extracted from the maps have been plotted as the following: mean value ± SE vs. concentration, and is reported in Figure S1 in Supplementary materials. In the saturated region, all of the active sites of the surface have been occupied by the molecules and, despite a higher or lower presence of randomly distributed hot spots that might affect the mean value, the signal maintained is stable. In the quantification region, the surface is progressively filled with molecules as a function of the increased concentration.

The reproducibility of the NPG-5h has been validated by repeating experiments in triplicates and by using each time a new sample and a fresh prepared solution. In the first and second trial, random spots on the surface have been collected; in the third trial, a map has been acquired. Figure S2a in Supplementary materials reports the mean ± SE of intensities for the trials at the concentration of 10^−12^ M, as a general example. In Figure S2b, the Analysis of the Variance (ANOVA) shows that if the three trials conducted with different pieces of NPG in the same concentration are comparable, then there are no significant differences in terms of the response of the substrate.

The LOD and EF of MGITC, using NPG-5h as the SERS substrate, was compared to other SERS substrates reported in the literature. Li et al. [44] developed a self-assembled plasmonic Au@Ag heterogeneous nano-cuboids (Au@Ag NCs) quantitative SERS sensor, which achieved an efficient SERS LOD of 8.7 × 10^−10^ M using MG as SERS analyte. Chen et al. [45] electrostatically assembled an organic framework material (MIL-100 (Fe)), and AuNPs as a SERS substrate, that displayed high sensitivity and good recyclability. The (MIL-100(Fe))-AuNPs revealed for MG a LOD of 10^−13^ M, based on the optimal substrate, and an EF of 7.67 × 10^7^. Huang et al. [46] proposed a nanostructured SERS substrate made up of a silicon pyramid array covered by a nanostructured gold film (AuNS @ SiPA) that shows the 0.01 nM LOD and EF 4.05 × 10^8^ for MG molecules sensing. Xu et al. [47] established a detection system for MG in aquaculture water. In this study, Ag nanoparticles were synthesized and dispersed on Au electrodes after centrifugation to produce a SERS active substrate. After optimizing pH values, preconcentration times and potentials, in situ detection SERS was then performed, allowing the analysis of low-concentrated MG and achieving the 2.4 × 10^−16^ LOD. Qiu et al. [48] investigated a nanoporous gold disk as a SERS activated substrate for the detection of MG, and found EF of 5.4 × 10^8^ with a LOD of 5 nM. Cheng et al. [49] investigated the electrostatic interaction between negatively charged AuNPs (gold nanoparticles) and positively charged COFs (covalent organic frameworks), producing AuNPs self-assembled on COFs (AuNPs@COFs) and achieving 6.2 × 10^−10^ M LOD and EF 5.3 × 10^5^. Wang et al. [50] studied the detection of MG on Ag nanodendrite substrates (AgNDs) synthesized by cyclic voltammetry electrodeposition on indium tin oxides; AgNDs showed excellent SERS activity with a detection limit of 9.4 × 10^−13^ and an enhancement effect of 5.4 × 10^9^. In a study by Zi et al. [51], magnetic Fe3O4 composite nanospheres coated with Au nanoparticles (Fe3O4@AuMCS) were synthetized and used as SERS substrate showing a LOD of 10−7 M with an estimated EF of ~1.10 × 10^5^. Kaminska et al. [52] fabricated an Au-coated GaN SERS surface using the photo-etched method and achieved an EF of 2.8 × 10^6^ for MG.

In the present work, NPG-5h substrate shows a very low limit of detection of 10^−16^ M and an EF of 7.9 × 10^9^. Among all the above-mentioned different substrates reported in literature, and collected in Table 2, NPG-5h performs better both in terms of LOD and EF.

The remarkable SERS enhancement of NPG-5h can be attributed to the nanoporous morphology of the sample surface that supplies an abundance of SERS-active sites, possibly related to the presence of grain boundaries and defects, facilitating the LSPR phenomenon. Furthermore, the creation of hotspots, due to the large curvatures of the nano-sized ligaments, as well as the electromagnetic coupling in the ligaments, also improved LSPR [53,54,55]. Our results indicate that this substrate is a promising tool for SERS detection of MGITC.

Considering that the environmental regulation of several industrialised countries poses the limit of 2 μg/L (i.e., 4 × 10^−9^ M) for MGITC in wastewaters [56], NPG-5h might play a substantial role in better monitoring the environmental pollution, owing to its remarkable ability of measuring the presence of MGITC even in extremely low concentrations. The substrate is, therefore, a promising tool for the detection of MGITC in real-world applications.

## 4. Conclusions

In conclusion, ultrasensitive detection of malachite green isothiocyanate (MGITC) via the SERS technique has been achieved, using a nanoporous gold substrate NPG-5h prepared via the chemical dealloying approach. The average ligament size of the as-dealloyed sample was estimated to be 65.0 ± 7.5 nm. Thanks to the presence of ligaments and nanosized pores on the NPG substrate surface, giving rise to an abundance of active sites; NPG-5h shows an outstanding SERS performance with high sensitivity via the LSPR phenomenon. A low limit of detection (LOD) of 10^−16^ M and a high-enhancement factor of 7.9 × 10^9^ have been achieved. The obtained LOD meets the EU regulatory requirements. The SERS analysis using the NPG-5h substrate proves to be a versatile and rapid detection for MGITC. It provides a new route for the development of the ultrasensitive detection of toxic effluents.

## Figures and Tables

**Table 1:** EDS analysis of NPG-5h.

at. %	Si	Cu	Pd	Ag	Au
Mean	1.0	5.3	0.3	0.1	93.3
SD	0.1	0.2	0.2	0.1	0.2

**Table 2:** Comparison of SERS performance of different substrates for the detection of MGITC.

SERS Substrate	Methods	Limit of Detection (LOD)	Enhancment Factor (EF)	References
Au@Ag NCs	SMG	8.7 × 10^−10^ M	-------	[44]
MIL-100(Fe)/Au	ST & EA	10^−13^ M	7.67 × 10^7^	[45]
Au NS @ SiPA	WE	1.0 × 10^−11^ M	4.05 × 10^8^	[46]
Ag NPs	EP	2.4 × 10^−16^ M	-------	[47]
NPG disk	NSL	10^−11^ M	5.49 × 10^8^	[48]
COF-AuNPs	EA	6.2 × 10^−10^ M	5.3 × 10^5^	[49]
Ag NDs	ED	4 × 10^−13^ M.	5.4 × 10^9^	[50]
Fe_3_O_4_@Au MCS	SMG & IR	10^−7^ M	1.1 × 10^5^	[51]
Au-GaN	PE	-------	2.8 × 10^6^	[52]
GO-AuNPs	RE	2.5 × 10^−6^ M	3.8 × 10^3^	[37]
(Ag/TiO_2_)	FTO	1 × 10^−12^ M	4.36 × 10^5^	[38]
NPG-5h	CD	10^−16^ M	7.9 × 10^9^	This work

NPs: Nanoparticles, NCs: Heterogeneous nanocuboids, NPG: Nanoporous gold, NS: nanostructured, NDa: Nanodome array, SiPA: Silicon pyramid array COF: Covalent organic frameworks NDs: nanodendrites, MCS: Magnetic composite nanospheres, MIL-100(Fe) (organic framework), GO-AuNPs: graphene oxide-gold nanoparticle hybrids, NSL: Nanosphere lithography, (Ag/TiO_2_): 3-D TiO_2_ nanorod scaffold coated with silver nanoparticles, ST: Solvent thermal synthesis and EA: Electrostatic assembly/interaction, SMG: Seed mediated growth, IR: Iterative reduction, WE: Wet etching, EP: Electrochemical preconcentration, CD: Chemical dealloying, ED: Electrodeposition. PE: Photo-etched, RE: reduction synthesis, FTO: fluorine-doped tin oxide.

## Data Availability

The raw/processed data required to reproduce these findings cannot be shared at this time, as the data also form part of an ongoing study.

## Supplementary Materials

The following supporting information can be downloaded at: https://www.mdpi.com/article/10.3390/ma16134620/s1, Figure S1. Intensity vs. concentration plot: the graph is divided in two colored regions. In cyan the saturated region and in orange the quantification region. Figure S2a The mean ± SE of intensities for the trials at the concentration of 10^−12^ M; Figure S2b the ANOVA (Analysis of the Variance) plot for the three trials.
