# Supplementary material for: Ultrasensitive Detection of Malachite Green Isothiocyanate Using Nanoporous Gold as SERS Substrate
Source: Materials (Basel). 2023 Jun 27;16(13):4620. doi: 10.3390/ma16134620 (PMC10342777; doi:10.3390/ma16134620)
Supplement: Supplementary file 1 [file materials-16-04620-s001.zip › materials-2445581-supplementary.pdf]

Ultrasensitive detection of malachite green isothiocyanate using nanoporous gold as SERS substrate

Deepti Raj[a], Noor Tayyaba[a], Ginevra De Vita[a], Federico Scaglione[a,*], Paola Rizzi[a]

[a]Dipartimento di Chimica e Centro Interdipartimentale NIS (Nanostructured Surfaces and Interfaces), Università di Torino, Via Pietro Giuria 7, 10125, Torino.
*Corresponding author: federico.scaglione@unito.it

Supplementary materials

Figure S1

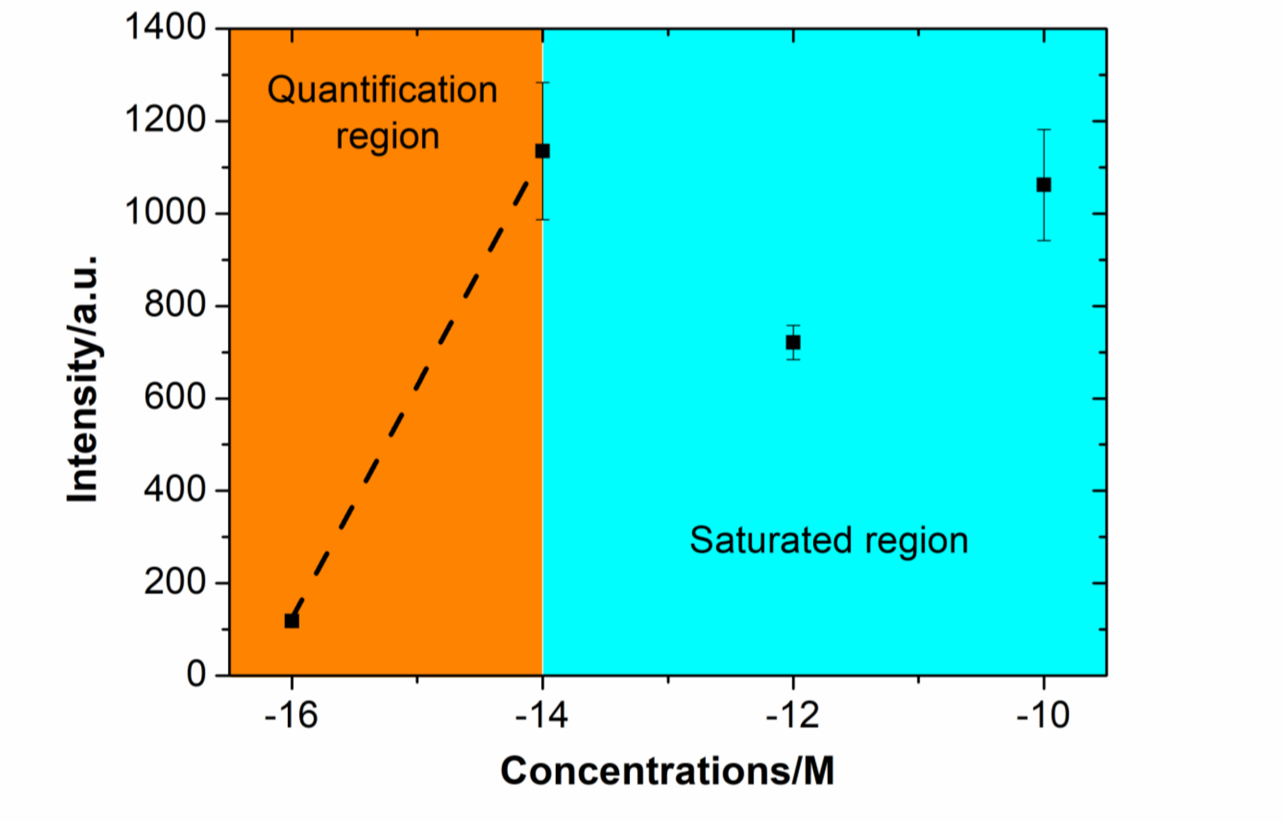

Figure S1: Intensity vs concentration plot: the graph is divided in two colored regions. In cyan the saturated region and in orange the quantification region. The dashed line is intended as a guide for the eyes.

Figure S2

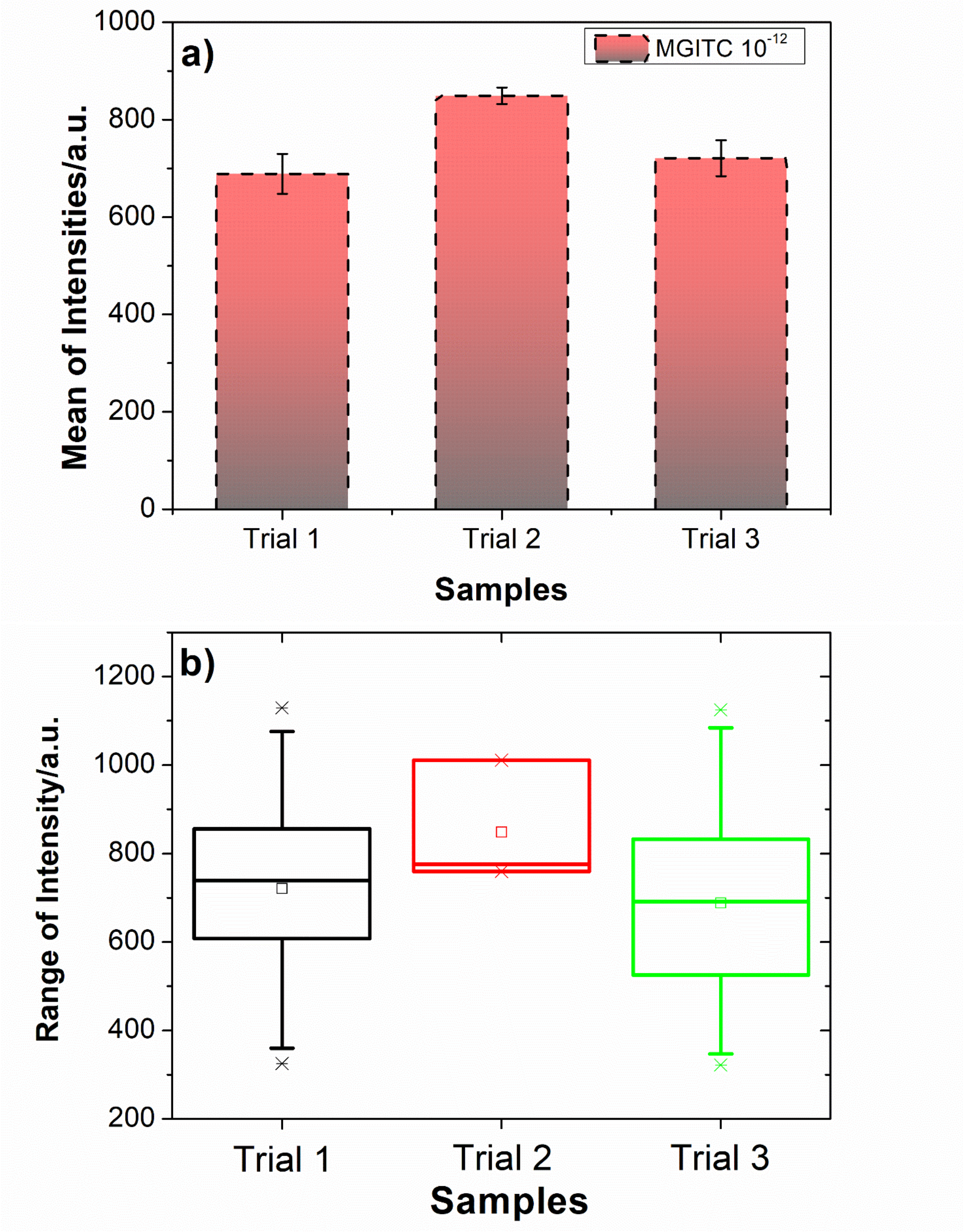

Figure S2: a) The mean ± SE of intensities for the trials at the concentration of 10^{-12} M; b) the ANOVA (Analysis of the Variance) plot for the three trials.
